# Trends in Single-Molecule Total Internal Reflection Fluorescence Imaging and Their Biological Applications with Lab-on-a-Chip Technology

**DOI:** 10.3390/s23187691

**Published:** 2023-09-06

**Authors:** Louis Colson, Youngeun Kwon, Soobin Nam, Avinashi Bhandari, Nolberto Martinez Maya, Ying Lu, Yongmin Cho

**Affiliations:** 1 Department of Systems Biology, Harvard Medical School, Boston, MA 02115, USA; louiscolson@g.harvard.edu (L.C.); avinashi_bhandari@hms.harvard.edu (A.B.); nolberto_martinezmaya@hms.harvard.edu (N.M.M.); ying_lu@hms.harvard.edu (Y.L.); 2 Department of Chemical Engineering, Myongji University, Yongin 17058, Republic of Korea; kye3526@mju.ac.kr (Y.K.); soobin019@mju.ac.kr (S.N.)

**Keywords:** single-molecule imaging, TIRF, fluorescence, data analysis, microfluidics

## Abstract

Single-molecule imaging technologies, especially those based on fluorescence, have been developed to probe both the equilibrium and dynamic properties of biomolecules at the single-molecular and quantitative levels. In this review, we provide an overview of the state-of-the-art advancements in single-molecule fluorescence imaging techniques. We systematically explore the advanced implementations of in vitro single-molecule imaging techniques using total internal reflection fluorescence (TIRF) microscopy, which is widely accessible. This includes discussions on sample preparation, passivation techniques, data collection and analysis, and biological applications. Furthermore, we delve into the compatibility of microfluidic technology for single-molecule fluorescence imaging, highlighting its potential benefits and challenges. Finally, we summarize the current challenges and prospects of fluorescence-based single-molecule imaging techniques, paving the way for further advancements in this rapidly evolving field.

## 1. Introduction

Imaging techniques provide powerful tools to visualize and quantify molecular interactions, cellular dynamics, and tissue architecture and are therefore instrumental in advancing our understanding of biological systems [1,2,3,4,5,6,7,8,9,10,11,12]. Certain imaging techniques can directly observe individual biomolecules such as oligonucleotides, proteins, and protein complexes. These single-molecule imaging techniques can provide information on the heterogeneity of the system which can often be difficult to determine using other methods. In recent years, single-molecule imaging with total internal reflection fluorescence (TIRF) has gained significant popularity due to its accessibility and high sensitivity in probing the properties of biomolecules. By enabling the visualization and tracking of individual molecules in exceptional spatial and temporal resolutions, TIRF-based single-molecule imaging has opened up new avenues for studying complex biological processes, including protein folding, protein–protein interactions, DNA replication, and cellular signaling [13,14,15,16,17,18,19,20,21,22].

In this review, we specifically explore in vitro single-molecule imaging with TIRF [11,23,24,25]. In addition to discussing the technical aspects of single-molecule imaging, this review surveys and highlights several exemplary applications of TIRF-based single-molecule imaging, especially microfluidic-based approaches. By showcasing the diversity of biological questions addressed using this technique, we aim to demonstrate its broad impact across various fields, including molecular biology, biophysics, and nanotechnology. Finally, we address the potential prospects and challenges of fluorescence-based single-molecule imaging techniques. We also discuss the limitations and potential sources of artifacts in single-molecule imaging experiments, as well as strategies to mitigate these issues.

## 2. Optical Systems for Single-Molecule Fluorescence Imaging

Fluorescence single-molecule imaging techniques rely on the utilization of optical radiation to probe individual molecules within a liquid or solid sample. To achieve successful single-molecule imaging, two key requirements must be met: (1) ensuring that resonant molecules are spatially resolved by the detector, and (2) providing a sufficient signal-to-noise ratio (SNR) for the single-molecule signal within a reasonable averaging time [22]. Consequently, a fundamental prerequisite for conducting single-molecule observations is to dilute the concentration of the target molecule of interest to exceedingly low levels (typically < 100 nM). The detection of single molecules via fluorescence-based methods demands careful optimization of the signal-to-noise ratio. Maximizing the signal requires the selection of an impurity molecule with the highest possible fluorescence quantum efficiency.

**Figure 1:**
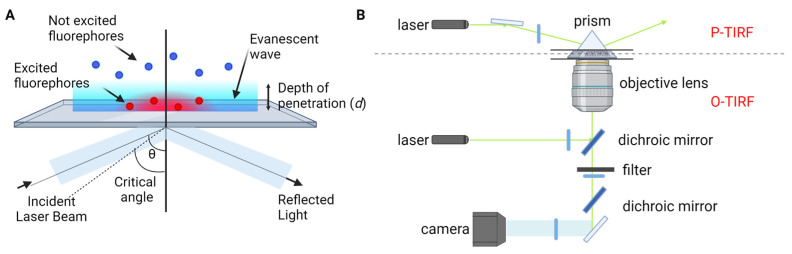
TIRF microscopy for single-molecule fluorescence imaging. (**A**) Principle of TIRF microscopy. (**B**) Types of TIRF microscopy: prism-type (P-TIRF) or objective-type (O-TIRF).

This approach harnesses recent advancements in fluorescence imaging techniques, including TIRF microscopy [23,24,25], super-resolution microscopy, and single-molecule localization microscopy [26,27,28,29,30,31,32]. TIRF microscopy, one of the most commonly employed tools in single-molecule fluorescence microscopy, capitalizes on the principle of total internal reflection. This phenomenon occurs when a laser beam strikes the interface between a medium with a higher refractive index (typically glass) and a medium with a lower refractive index (such as a sample solution) at an angle greater than the critical angle (Figure 1A). As a result, an evanescent wave is generated, which excites fluorophores in the immediate vicinity of the interface, facilitating the visualization of single molecules near the sample surface. TIRF microscopy is practically implemented by using either a quartz prism or the microscope objective to generate the evanescent field and illuminate surface-immobilized molecules (Figure 1B). TIRF microscopy offers exceptional optical sectioning and background suppression, leading to a high signal-to-noise ratio.

The evanescent field intensity, I(z), at a perpendicular distance z from the interface is described by Equation (1).

(1) $$I\left(z\right)=I\left(0\right)\exp(-\frac{z}{d}),$$

(2) $$d=\frac{\lambda_{0}}{4\pi }(n_{1\;}^{2}{sin}^{2}\theta -n_{2}^{2}{)}^{-1/2},$$

where $I\left(0\right)$ represents the evanescent field intensity at the interface (Figure 1A). The characteristic penetration depth (Equation (2)), *d*, is determined by the wavelength of incident light ($\lambda_{0})$, refractive index of the medium through which the light initially passes ($n_{1}$) and in contact with the sample ($n_{2}$), and incident angle (θ). Typically ranging between 30 and 200 nm, the penetration depth defines the region within which fluorophores are effectively excited by the evanescent wave.

In addition, the detection of individual fluorophores is a critical aspect of single-molecule fluorescence imaging. Here, the numerical aperture (NA) is one of the key parameters. High NA objectives are commonly used to maximize light collection and detection efficiency. The specific NA value depends on the imaging setup and the desired resolution and sensitivity. For conventional single-molecule fluorescence imaging, objectives with NA values ranging from 1.2 to 1.49 are frequently employed. These objectives offer a balance between high light collection efficiency and reasonable working distances. They are suitable for imaging samples in various configurations, including liquid solutions, solid surfaces, and biological specimens.

The choice of camera is another important factor in the detection of individual fluorophores. Ultimately, digital cameras capture the photons from individual fluorescent molecules and convert the light into electrical signals. The cameras used for single-molecule TIRF imaging tend to have quantum efficiencies above 80%, spectral range between 300 and 1100 nm, low readout noise, and millisecond readout speeds [33]. Electron multiplying charge coupled devices (EMCCDs) and the scientific complementary metal–oxide–semiconductor (sCMOS) devices are the most common types of cameras used in single-molecule imaging. Note that most of the biological applications discussed in Section 5 use an EMCCD camera. Some recent laboratory advances in imaging technology may further improve the performance of scientific cameras across the broadband spectrum [34,35,36].

## 3. Sample Preparation

**Figure 2:**
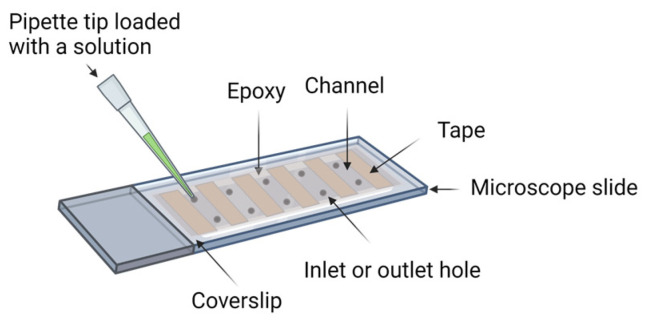
Schematic representation of an imaging device. The construction of an imaging device entails the integration of a microscope slide and a coverslip, employing double-sided tape for precision juxtaposition, followed by hermetic sealing with epoxy resin. The holes on the slide are used as the inlet and outlet for solution exchange.

Sample preparation is a critical step in single-molecule imaging of biological molecules for repeatable and reliable results. In this section, we define “sample preparation” as the preparation of the imaging device (Figure 2) and any labeling of the biological molecules. Generally, biomolecules non-specifically adhere to the surfaces of the imaging device. Thus, the preparation of the imaging device includes a passivation step to reduce non-specific binding and false-positive signals. Fluorophores provide a readout for interactions between molecules or the functions of the reaction system. Preparing biological molecules for the experiments includes a labeling step to conjugate fluorophores to molecules of interest and a strategy to constrain the location of the molecules of interest in the imaging region [37,38]. There are methods that detect freely diffusing single molecules [39,40], but in this review, we limit our scope to single-molecule strategies with immobilized molecules.

### 3.1. Surface Passivation

**Figure 3:**
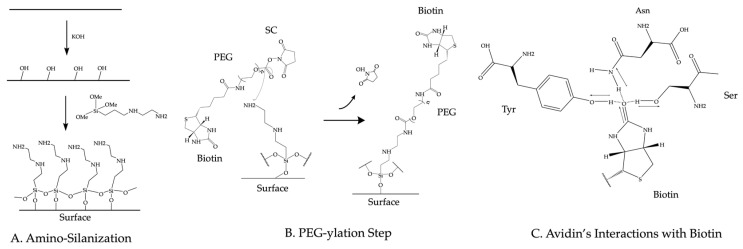
Chemical diagrams of surface preparation steps. (**A**) Diagram of amino silanization of glass surface. (**B**) Diagram of PEG-ylation step with heterobifunctional biotin–PEG–succinimidyl carbonate (SC) ester. (**C**) Diagram of the interactions between avidin and biotin.

Established passivation techniques to prepare the imaging device rely on coating chemically treated glass surfaces with biocompatible reagents such as polyethylene glycol (PEG), phospholipids, or Tween-20. In this section, we briefly describe the PEG, lipids, and Tween-20 passivation methods. These methods produce similar results and are described in depth elsewhere [41,42,43]. The PEG passivation method relies on the amino-silanization of the glass surface. Usually, researchers use KOH to form alcohol groups on the glass surface. Amino silane can react with these alcohol groups on the surface. This reaction (shown in Figure 3A) takes the following form: $ROH+{\left(OCH_{3}\right)}_{3}SiOCH_{3}\to CH_{3}OH.$ To complete the passivation, commercially available PEG ester molecules react with the silane groups (Figure 3B) to PEGylate the surface of the coverslip [41]. This covalent passivation method can withstand harsh protein denaturing conditions such as 8 M guanidinium chloride (GdmCl) [44] and 4M urea [45]. For the Tween-20 passivation, a dichlorodimethylsilane (DDS)-treated glass surface forms a hydrophobic coating that can be passivated with the addition of the biocompatible surfactant Tween-20 [42]. For the passivation with phospholipids, glass devices can be incubated with liposomes to form a fluid lipid bilayer. The liposomes used for passivation in single-molecule TIRF experiments have been made from lipids such as 1,2-dioleoyl-sn-glycero-3-phosphocholine (DOPC) and egg phosphatidylcholine [46,47].

### 3.2. Surface Functionalization

Typically, surface-immobilized avidin, streptavidin, or neutravidin (from here on avidin will be used interchangeably with any of these forms of avidin) tether biotinylated biomolecules to the imaging region. To immobilize avidin on the surface, biotins are introduced onto the surface before or during the passivation step. For the PEG passivation method, a percentage of the PEG molecules that passivate the surface contain a biotin moiety on the opposite end from the ester group [43]. The ester group in the biotinylated PEG molecule reacts with the amine group on the surface as shown in Figure 3B. For the Tween-20 passivation method, researchers introduce biotinylated BSA to the imaging device prior to passivation. The biotinylated BSA adheres to the hydrophobic surface before Tween-20 passivates the surface [42]. For the lipid passivation method, either a fraction of the lipids will be biotinylated [46] or avidin will be directly applied to the imaging surface before passivation [47]. Avidin binds to biotin molecules with high affinity (K_D_~10^−15^ M) [48]. This affinity comes from a number of hydrogen bonds formed between the amino acids of avidin’s biotin-binding site and the biotin molecule as diagramed in Figure 3C. Since avidin forms a tetramer, avidin molecules bound to the biotins on the imaging surface still contain available biotin binding sites to immobilize biotinylated molecules.

### 3.3. Protein Biotinylation

**Figure 4:**
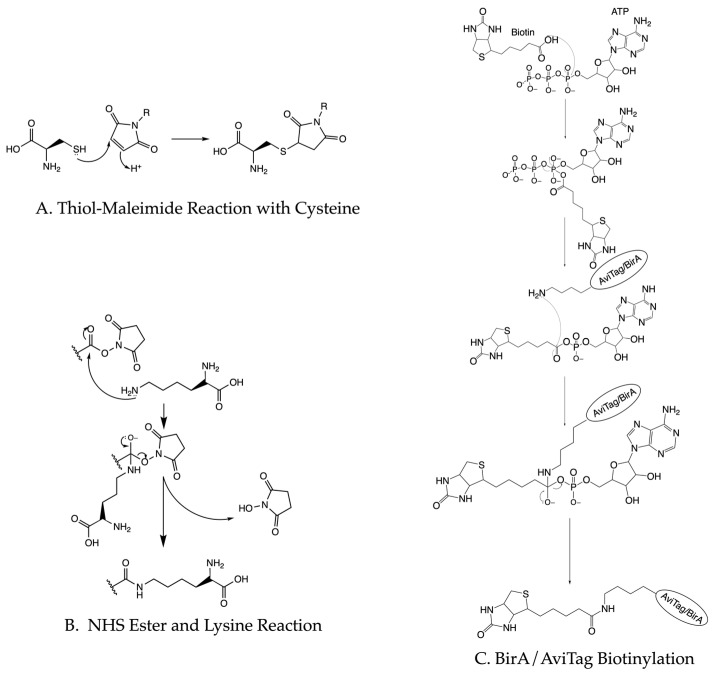
Chemical diagrams of common protein labeling strategies. (**A**) Thiol–maleimide reaction represents a common way to label cysteine residues in proteins. (**B**) NHS ester reaction with lysine provides a way to label proteins. (**C**) The process of AviTag biotinylation as a way to tether proteins to immobilized avidin.

One strategy to directly biotinylate proteins of interest involves introducing an AviTag [49,50,51]. The BirA ligase recognizes the 15 amino acid AviTag and conjugates biotin to the tag’s only lysine residue [52,53]. BirA biotinylation occurs through a two-step reaction where biotin first reacts with ATP before the amine group of AviTag’s lysine residue attacks the ester in biotin (Figure 4C). Overexpressing BirA ligase can biotinylate the protein in vivo [53] or purified BirA can biotinylate the protein in an in vitro reaction system [53,54]. Other direct coupling methods include using biotinylated peptides [55] or ligating a biotinylated peptide to the C-terminus of a protein of interest [14]. Biotinylated peptides or molecules with a high affinity to fusion tags can also be used to introduce biotin to the proteins of interest [56]. Biotinylated antibodies [57,58] or biotinylated nucleotide oligos [59,60,61] can indirectly couple proteins to the surface. Tethering the sample to the imaging surface does not require high efficiency. The vast majority of unbiotinylated molecules will not stick to the passivated surface and at saturating conditions, most biotin-binding sites will bind to biotinylated biomolecules.

### 3.4. Protein Fluorescence Labeling

The requirements for fluorescent labeling are more stringent. One of the most common strategies conjugates fluorescent molecules with maleimide groups onto the thiol groups on the cysteines of proteins (Figure 4A). This strategy works best for small proteins without essential cysteine residues. Similarly, lysine can react with NHS ester groups on fluorophores (Figure 4B). Recent studies also use unnatural amino acids to perform click chemistry with fluorophores [54,62]. This method works well for large proteins or protein complexes and for proteins that harbor essential cysteine residues. Additionally, the development of high-affinity protein fusion tags allows N- or C-terminal fusion tags to provide a specific and simple way to bind a fluorophore to the target of interest [63]. Fluorescently labeled antibodies offer a potential alternative to these aforementioned methods. For all of these, the choice of fluorophores and the labeling strategy will depend on the biological application. Roy et al. [33] provide a practical overview of the TIRF-based single-molecule experiments with Förster Resonance Energy Transfer (FRET).

## 4. Analysis Methods

**Figure 5:**
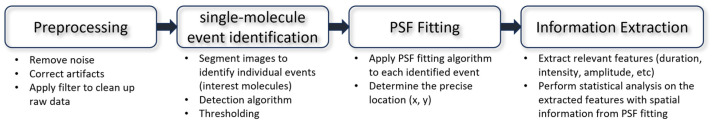
Workflow of single-molecule imaging data analysis.

Single-molecule data often exhibit inherent noise stemming from both the system under study and the measurement instrument. This noise can manifest in various forms, including sample stage drift [64,65], Gaussian fluctuations [66,67], non-Gaussian variations [68,69,70], diffusive behavior [71,72], and even undefined sources [73,74]. Complications arise particularly when the nature of the underlying fluctuation is unknown, as it can potentially follow either a Gaussian or non-Gaussian distribution. Consequently, extracting meaningful information from single-molecule data poses significant challenges. In this section, we will introduce key steps in the analysis with key examples (Figure 5).

### 4.1. Point Spread Function Fitting

To achieve high spatial resolution, it is essential to precisely localize the position of each detected fluorophore. This localization is typically performed using a technique called point spread function (PSF) fitting, where the observed intensity distribution of a fluorophore is fit to a mathematical model of the PSF. By accurately determining the center of the PSF, the position of the fluorophore can be determined with sub-pixel precision, enabling precise localization of single molecules. Sage et al. [29] comprehensively evaluated software packages for single-molecule localization microscopy (SMLM). Many modules from these software packages would be usable for TIRF-based single-molecule fluorescence imaging datasets.

### 4.2. Extracting Information from Signal

Among the most prevalent types of single-molecule imaging data are time series signals characterized by values ranging from zero to an upper limit. To extract the desired information, several techniques have been developed to fit the noisy time series data to an idealized model involving discrete steps and dwell times [73]. One widely employed method is hidden Markov modeling (HMM) [75,76,77,78,79]. HMM enables the identification of hidden (unobservable) states within a Markovian process, where the present and future states depend solely on the current state, independent of the system’s prior states. The idealized model from HMM is a reliable way to extract the FRET states, dwell times, and rate constants from single-molecule time series data.

### 4.3. Deep Learning

Deep learning has emerged as a powerful tool for analyzing single-molecule fluorescence imaging data, particularly for handling large volumes of complex and noisy data [80,81]. Specifically, deep learning algorithms, such as convolutional neural networks (CNNs) and recurrent neural networks (RNNs), excel in recognizing patterns and features in images that may be challenging for manual identification. CNNs are well-suited for detecting and localizing individual molecules, while RNNs can analyze the temporal dynamics of fluorescence signals. Deep learning can also extract more intricate information from single-molecule data, including classifying molecular states based on fluorescence properties or predicting molecular interactions. Nonetheless, challenges exist, such as the need for large, annotated datasets, which can be time-consuming and costly to generate, as well as the risk of overfitting or underfitting models, potentially leading to inaccurate or unreliable results. Liu et al. summarized the deep learning application in single-molecule analysis [80].

## 5. Biological Application

Single-molecule imaging of in vitro systems proves to be a powerful tool for researching conformational dynamics, protein folding, protein modifications, and protein interactions. Results from single-molecule imaging investigations can lead to important insights into biological processes such as transcription [60,61], protein synthesis [82,83], and protein degradation [13,54,63]. Single-molecule methods offer unique insights into the heterogeneity of the sample. Observing the fluorescence of biomolecules in vitro further offers the benefit of time scales from milliseconds to minutes, location of the biomolecules within the system, tight control over the components in the system, and potential readouts for conformational states. In this section, we describe some of the recent applications of single-molecule TIRF imaging.

### 5.1. Conformation Dynamics

Elucidating protein conformational dynamics often reveals important mechanistic insights into how proteins function. To observe conformational dynamics in real-time, researchers can combine single-molecule TIRF with fluorescent resonance energy transfer (FRET) [33]. FRET experiments estimate the efficiency of energy transfer, *E*, as described by Equation (3).

(3) $$E={\left(1+{\left(\frac{r}{R_{0}}\right)}^{6}\right)}^{-1},$$

In Equation (3), r represents the distance between one donor fluorophore and one acceptor, and $R_{0}$ represents the Förster radius for a specific donor–acceptor pair at which E=0.5 [33]. To illustrate typical values for $R_{0}$, the Förster radii for Cy3-Cy7, Cy3-Cy5, and Cy5-Cy7 are 3.8 nm, 5.4 nm, and 6.2 nm, respectively [84]. The apparent FRET efficiency and corrected FRET efficiency are calculated from the intensity of the fluorophores using Equations (4) and (5).

(4) $$E_{app}=\frac{I_{A}}{I_{A}+I_{D}},$$

(5) $$E_{corrected}={\left(1+\gamma \frac{I_{D}}{I_{A}}\right)}^{-1},$$

**Figure 6:**
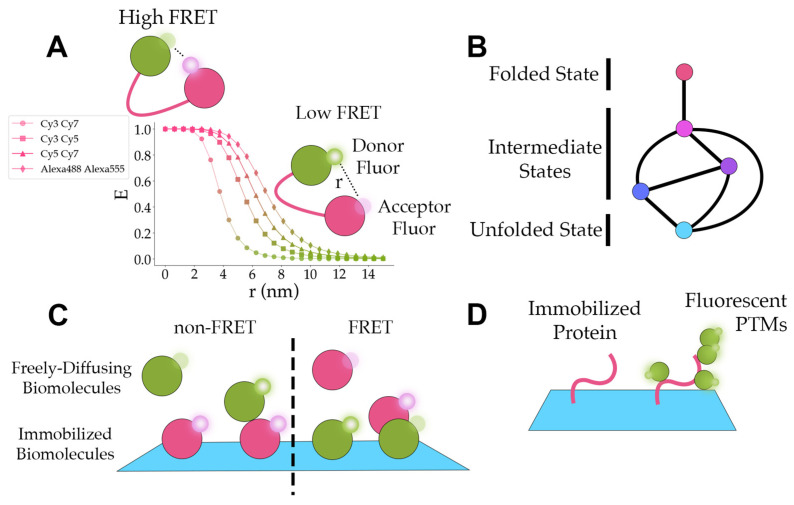
Application concepts. (**A**) A graph of ideal smFRET efficiencies (*E*) versus distance between fluorophores (r) for different fluorophore pairs. The Förster radii for Cy3-Cy7, Cy3-Cy5, Cy5-Cy7, and Alexa488-Alexa555 are 3.8 nm, 5.4 nm, 6.2 nm, and 7 nm, respectively [84]. The green and pink colors distinguish between domains labeled with donor and acceptor fluorophores. The small, semi-transparent circles represent fluorophores that are not radiating light. The small circles with white highlights represent fluorophores that are emitting light. (**B**) A diagram displaying the paths (the lines connecting the circles) between folded, intermediate, and unfolded states (the circles). The different colors illustrate the distinct smFRET efficiency peaks for each state. (**C**) A representation of freely diffusing fluorescent biomolecules interacting with immobilized fluorescent biomolecules. In the non-FRET case, green and pink colors separate biomolecules with spectrally distinct fluorescent signals. In the FRET case, pink biomolecules contain an acceptor fluor and green biomolecules contain a donor fluor. (**D**) A diagram of an immobilized protein (pink) and an immobilized protein with fluorescent post-translational modifications (PTMs) (green).

In Equations (4) and (5), $I_{A}$ represents the intensity of the acceptor fluorophore and $I_{D}$ represents the intensity of the donor fluorophore. In Equation (5), γ represents the correction factor [33]. The observed apparent FRET efficiency can be affected by dye orientation, dye conjugation, and instrument factors, and thus only provides an approximation for the distance between donor and acceptor dyes [33]. Nevertheless, by conjugating one acceptor fluorophore and one donor fluorophore at carefully picked locations on a protein of interest, FRET efficiencies can correspond to distinct conformational states (Figure 6A). In the last three years, smFRET has been applied to uncover some of the conformational dynamics of proteins such as CRISPR (clustered regularly interspaced short palindromic repeats)-Cas9 (CRISPR-associated protein 9) from *Streptococcus pyogenes* [51], mouse metabotropic glutamate receptor 2 (mGluR2) [62], and the yeast 26S proteasome [54].

The CRISPR-Cas9 RNA-guided endonuclease enzyme performs multiple steps and conformational changes to cleave DNA [51]. In particular, the HNH and RuvC nuclease domains on CRISPR-Cas9 cleave the target strand (TS) and nontarget strand (NTS) of DNA, respectively [51]. To characterize the post-catalytic conformational changes in the HNH domain with respect to the TS, Wang et al. developed a Cas9 construct with a single cysteine residue on the HNH domain (Cas9_LD750_) [51]. Because the TS is relatively stationary, labeling the TS with Cy3 and the HNH domain with LD750 provided a highly sensitive reporter for the conformational changes in the HNH domain [51]. Choosing LD750 rather than Cy5 as the acceptor increases the sensitivity of the FRET efficiency at shorter distances. Using this smFRET reporter with catalytically dead RuvC and HNH variants, they found that only variants that could cleave the TS showed fluctuations between FRET efficiencies (*E*) ~0.38 and ~0.61 [51]. Ultimately, this single-molecule fluorescence reporter provided direct support for the high flexibility of the HNH domain post-DNA cleavage.

In addition to elucidating conformational dynamics, smFRET is also used to characterize the propagation of conformational changes. Activation of metabotropic glutamate receptors (mGluRs) through binding the excitatory neurotransmitter L-glutamate results in local and global conformational changes that propagate through the ligand-binding Venus flytrap (VFT) domain, cysteine-rich domain (CRD), and 7-transmembrane (7TM) domain to reach the intracellular G protein-binding interface [62]. To construct a smFRET reporter on the CRD conformational changes, Liauw et al. incorporated an unnatural amino acid at amino acid 548 in the CRD of the mouse mGluR2 [62]. A copper-catalyzed azide–alkyne click reaction labeled the proteins with either Cy3 or Cy5 and labeled mGluR2 with a C-terminal FLAG-tag were immobilized in the imaging region with biotinylated FLAG antibodies. These smFRET studies uncovered that the CRD domain dynamically transitions between two intermediate FRET states (*E* ~0.51 and *E* ~0.71) and two FRET states corresponding to the inactive (*E* ~0.31 and predominant population in the absence of an agonist) and active states (*E* ~0.89) of mGluR2 [62]. Further, they labeled a glutamate-binding defective monomer with an N-terminal SNAP-tag and BG-ATTO488 (SNAP-tag substrate) fluorophore to determine that heterodimers predominantly reside in the *E* ~0.51 [62]. Their findings provide evidence that shows mGluR activation proceeds through multiple states including one state where one of the ligand binding domains is inactive.

Similarly, Jonsson et al. established, using an unnatural amino acid incorporation strategy, the first single-molecule observations of the conformational changes between the engagement-competent s1 state and the processing-competent non-s1 states of the large, multi-subunit ~2.5 MDa yeast 26S proteasome [54]. The distance between the subunit Rpn9 and the N-terminal of subunit Rpt5 on the 26S proteasome decreases by ~3 nm when the 26S proteasome goes from the s1 state to the non-s1 state. By labeling Rpn9 with LD555 and Rpt5 with LD655, the s1 state corresponded to a distinct smFRET *E* of ~0.3 and the non-S1 state corresponded to a distinct E of ~0.75 [54]. Using this system, they observed that the 26S predominantly resided in the low FRET s1 state [54]. The addition of substrate biased the 26S proteasome to the non-S1 state [54]. Substrates with higher thermodynamic stability increased the frequency of high FRET non-S1 states to return briefly to the low FRET s1 state [54]. Furthermore, the presence of tetra-ubiquitin chains allosterically stabilized the s1 state and reduced the rate of the s1-to-non-s1 transition by ~3-fold, suggesting ubiquitin chain binding to the 26S proteasome might promote substrate engagement and degradation initiation [54]. In these examples, researchers leveraged the advantages of single-molecule fluorescence imaging to characterize conformational dynamics, new conformational states, and allosteric regulators.

### 5.2. Protein Folding/Unfolding

Single-molecule fluorescence experiments can identify distinct intermediate states and characterize the transition paths between each of the states during folding or unfolding (Figure 6B). Free-diffusion single-molecule experiments ameliorate concerns of artifacts from immobilizing a protein but limit the time of observation to milliseconds. To increase the observation time scale and mimic free-diffusion conditions, Pirchi et al. encapsulated adenylate kinase in a biotinylated lipid vesicle and tethered the lipid vesicle to the surface through biotin–streptavidin–biotin–PEG surface interactions [46]. The average FRET efficiency from the lipid vesicle-constrained single-molecule experiments showed agreement with the FRET efficiency from bulk measurements and free-diffusion single-molecule experiments [46]. They found the unfolding/folding of adenylate kinase involves at least six states with an average trajectory length of 4.6 s and higher concentrations of denaturant increase the probability of sequential transitions [46].

### 5.3. Protein Interactions

One of the more intriguing applications of single-molecule fluorescence experiments is the detection of protein interactions (Figure 6C). This provides information on the dynamics, the interaction’s dependence on the system, and the interaction’s effects on the function of proteins. Bibeau et al. demonstrated that yeast cofilin binds to actin filaments independent of curvature, but their results suggest actin curvature may facilitate cofilin dissociation [85]. They also show that cofilin clusters grow asymmetrically with the growth towards the pointed end of the actin being twice as fast as the growth rate towards the barbed end [85]. These results offered novel insights into cofilin interactions with actin and cofilin clusters under different conditions.

Asher et al. [57] observed the dynamics between a model GPCR protein, human V_2_ vasopressin receptor, and β-arrestin 1. They show that the β-arrestin 1 C-terminal tail binds to its own N-terminal positively charged groove to block interaction with the phosphorylated C-terminal of the human vasopressin receptor [57]. Immobilized β-arrestin 1 was labeled with LD555p and LD655 to directly observe the distance of β-arrestin 1′s C-terminal tail to the N-terminal groove. Alone, β-arrestin 1 demonstrates a stable high FRET state indicating interactions between the C-terminal and N-terminal groove. The addition of phosphomimetic C-terminal peptides from human vasopressin receptors transitioned the high FRET states to a lower FRET state indicating displacement of the C-terminal and N-terminal groove. A full-length chimera receptor protein when bound with the agonist epinephrine also demonstrated transitions to a lower FRET state albeit with shorter dwell times in the lower state.

Poyton et al. investigated the interactions between nucleosomes and chromatin remodeler, SWR1 to understand the timing of histone and DNA dynamics when SWR1 mediates histone H2A exchange for H2A.Z [59]. They find that most SWR1 binding events do not lead to H2A exchange [59]. However, when exchange occurs, H2A remains in complex with SWR1–nucleosome complex for tens of seconds after being displaced and DNA rewrapping takes about 1.4 s.

To understand the mechanism of the drug ataluren on the eukaryotic ribosome, Huang et al. labeled the peptide and tRNA. They found that ataluren binds to the ribosome and competes with the release factor complex (RFC) [86]. In the absence of ataluren, 20 nM of RFC resulted in a 50% maximum effect on peptide and tRNA dissociation with an effective concentration (EC50) of 20 nM [86]. With the ataluren concentration at 1000 uM, the EC50 for RFC increases to 100 nM. This indicates that ataluren plays a role in regulating RFC activity and alters the dissociation of peptides and tRNA from the ribosome.

Roca et al. used single-molecule fluorescence imaging to investigate the binding of small RNA to the RNA-binding protein Hfq [49]. The content of the small RNA and the binding interface on Hfq determined the effectiveness of the small RNA binding to Hfq [49].

### 5.4. Protein Post-Translational Modifications

Post-translational modifications of proteins regulate the activity and destruction of proteins in the cell. Single-molecule in vitro fluorescence imaging can be used to directly observe the addition of post-translational modifications as in Figure 6D or elucidate the effects of post-translational modifications on the system. Recently, this technique has been applied to studying ubiquitination [13,50] and phosphorylation [57].

Branigan et al. directly observed that ubiquitin transfer proceeds from a high FRET signal corresponding to the closed ring conformational state of the E2 ubiquitin ligase [50]. Lu et al. elucidated the dynamics of ubiquitination by the E3 ligase APC, where APC displays a biphasic transfer to substrates [13,14]. Initially, APC adds three to five ubiquitins to the substrate within the first five seconds. After, APC slowly elongates the ubiquitin chains. The results from single-molecule fluorescence imaging of in vitro systems provided critical information on protein structural states, protein interactions, and protein modifications that are difficult to obtain any other way. The investigations highlighted in this section are summarized in Table 1.

## 6. Application of Lab-on-a-Chip Techniques for Single-Molecule Fluorescence Imaging

Lab-on-a-chip techniques have emerged as valuable tools in single-molecule fluorescence imaging, offering numerous advantages and enabling new possibilities for experimental design and analysis [87,88,89,90,91,92,93]. Lab-on-a-chip devices provide unparalleled control and manipulation capabilities, enabling precise management of fluid flow and sample handling in single-molecule imaging experiments through the utilization of a laminar flow regime. The controlled flow within microfluidic channels not only facilitates efficient sample processing but also aids in reducing background noise by removing unbound or non-specifically bound molecules, thereby enhancing the SNR. Moreover, lab-on-a-chip platforms leverage miniaturized channels and chambers to create controlled microenvironments for the delivery, mixing, and incubation of samples, as well as the manipulation of individual molecules. In this section, we will provide a concise introduction to several key lab-on-a-chip-based platforms and highlight their recent applications in biological research [94]. These platforms have successfully overcome the limitations of single-molecule imaging while also improving experimental modalities and data quality.

### 6.1. Enhance Signal-to-Noise Ratio

**Figure 7:**
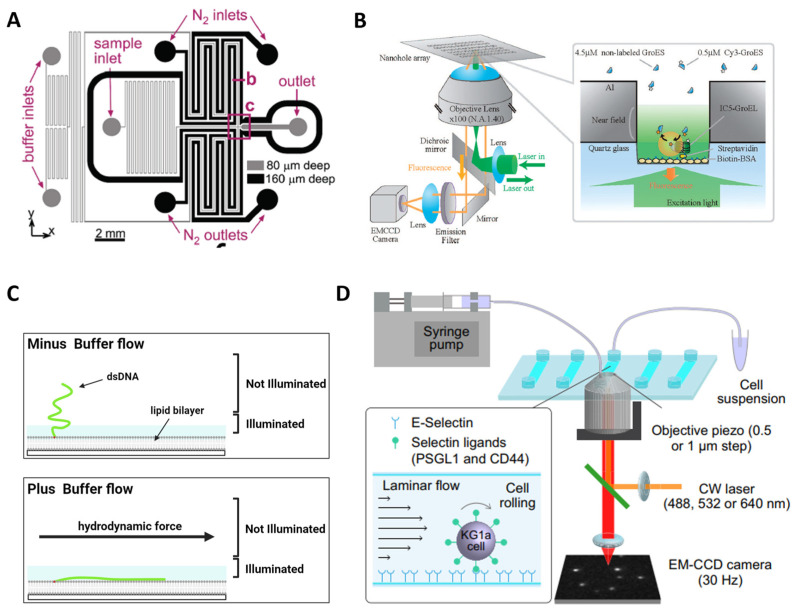
Examples of lab-on-a-chip applications for single-molecule fluorescence imaging. (**A**) Photobleaching is reduced by deoxygenation via gas diffusion through porous channel walls in a microfluidic device. Reprinted with permission from Ref. [98]. Copyright 2009 American Chemical Society. (**B**) Real-time imaging of single-molecule fluorescence with a ZMW for the study of protein–protein interaction. (**C**) Microfluidic-based DNA curtain platform allows parallel data acquisition of individual protein−DNA interactions in real time. Adapted with permission from Ref. [104]. Copyright 2008 American Chemical Society. (**D**) A microfluidics-based single-molecule live cell fluorescence imaging platform for the study of spatiotemporal dynamics of selectin–ligand interactions during cell rolling.

One advantage of single-molecule imaging over ensemble studies is its superior time domain resolution for investigating molecular dynamics. However, this advantage can be compromised by the limited photostability of singlet exciton emission, which is prone to bleaching and blinking due to factors like O_2_ and intersystem crossing. The stochastic fluctuations resulting from blinking are unrelated to the underlying biological behavior. To overcome these challenges, microfluidics has been employed by incorporating oxygen scavengers and triplet quenchers into the imaging buffer [95,96]. By carefully designing the setup, this approach has recently facilitated the shortest observation durations [97]. It effectively addresses the limitation of time domain resolution posed by fluidic speed, particularly during fluidic mixing. A different method was shown [98], wherein the imaging channels were integrated with those consistently supplied with nitrogen ventilation (Figure 7A). Furthermore, sophisticated microfluidic architectures can reduce flow velocities immediately after mixing, enabling longer optical interrogations [99].

### 6.2. Increase Sample Concentration

As mentioned, single-molecule fluorescence imaging techniques are limited to using pico- to nanomolar concentrations to ensure that only single molecules are resonant within the laser-probed volume and provide a sufficient SNR. However, many biologically relevant processes occur at micromolar level concentrations, necessitating a reduction in the conventional observation volume by three orders of magnitude. Here, arrays of zero-mode waveguides (ZMWs) consisting of subwavelength holes in a metal film provide a means to increase sample concentrations to the micromolar range while confining the observation volume to zeptoliter dimensions (Figure 7B) [100,101,102]. This breakthrough enables studies in the physiological concentration range and has been successfully applied in real-time, protein–protein interactions [102]. ZMWs have also been utilized to investigate ribosome-mediated translation processes, allowing the observation of tRNA transit in real-time at physiological concentrations [103]. Additionally, ZMWs have demonstrated versatility in studying biomolecular interactions, protein receptor diffusion, and oligomerization on living cell membranes [94].

### 6.3. On-Chip Single-Molecule Manipulation

On-chip devices have been developed with the capability to spatially modulate individual molecules with nanometer or even sub-nanometer sensitivities. A notable example is the microfluidic-based “DNA curtain”, which has recently emerged as an elegant on-chip tool for investigating DNA–protein interactions [16,17,18,47,104]. Illustrated in Figure 7C, this technique involves driving DNA molecules that are tethered to a fluidic lipid bilayer on the surface. These molecules drift downstream under the influence of flow until they encounter a thin layer of metal, which serves as a diffusion barrier. Consequently, the DNA molecules align with each other, forming what is referred to as a DNA curtain [47]. This fluidic-chip setup has proven highly successful in unraveling the searching modes of a DNA repair complex at DNA damage and elucidating the disruption of a transcription complex by a DNA translocase at the single-molecule level [18]. In addition, Alwan et al. utilized a microfluidics-based single-molecule live cell fluorescence imaging to study the spatiotemporal dynamics of selectin ligands on the membrane tethers and slings during cell rolling (Figure 7D) [105].

### 6.4. Microenvironment Control

Lab-on-a-chip technology also affords precise control over the microenvironment surrounding single molecules. Variables such as temperature, pH, and chemical gradients can be precisely manipulated within microfluidic devices, providing valuable insights into the impact of different conditions on the behavior and functionality of biomolecules. This level of control allows for the investigation of dynamic processes under various physiological or pathological conditions, mimicking complex biological environments. For example, Zhang et al. studied the in situ conformational response of single biomolecules such as DNA to a change in environmental solution conditions [106]. This level of control allows researchers to probe biomolecular interactions, enzymatic activities, and other dynamic processes with exceptional temporal resolution.

Moreover, lab-on-a-chip devices possess the remarkable capability of automation when integrated with other techniques. This integration not only minimizes experimental bias but also facilitates high-throughput screening, data acquisition, and analysis, which are indispensable for conducting large-scale single-molecule studies. By automating microfluidic processes, researchers can streamline their experiments, achieve consistent and reliable results, and analyze vast amounts of data efficiently.

## 7. Conclusions and Future Directions

Despite the remarkable advancements in fluorescence-based single-molecule imaging techniques, several limitations still exist, which present opportunities for further development and improvement. One of the major challenges in single-molecule imaging is photobleaching, which refers to the irreversible loss of fluorescence caused by repeated excitation. This phenomenon poses limitations on the observation time and hampers the investigation of long-lived biological processes. Another critical aspect of precise single-molecule imaging is the efficient and specific labeling of biomolecules with fluorophores. However, existing labeling methods may introduce artifacts, alter the natural behavior of molecules, or impact their functionality. Future investigations should aim to enhance labeling techniques, striving for high efficiency, specificity, and minimal disruption to the biological system at hand. This pursuit encompasses developing novel labeling strategies, including genetically encoded tags and chemical modification approaches, which afford improved targeting capabilities. Simultaneous imaging of multiple molecular species or different structural components within complex systems holds immense value. However, spectral overlap among fluorophores presents challenges in reliable multi-color imaging. Future endeavors involve designing and synthesizing fluorophores with narrower emission spectra and refining spectral separation techniques. Additionally, the development of advanced imaging setups, detection algorithms, and novel fluorophore combinations will enable more precise and efficient multi-color imaging experiments.

Furthermore, while fluorescence-based single-molecule imaging offers impressive spatial and temporal resolution, advancements are sought to observe dynamic molecular processes at an even finer scale. Innovations in super-resolution techniques like SMLM or stimulated emission depletion (STED) microscopy can push the boundaries of spatial resolution [27,28,29,31,107,108,109,110,111]. Similarly, the progress in ultrafast imaging methods and the design of detectors with heightened sensitivity and speed will facilitate the study of rapid molecular dynamics with heightened temporal resolution.

As single-molecule imaging experiments generate increasingly complex and voluminous datasets, there arises a need for sophisticated data analysis techniques and integration with other omics data. Future investigations should concentrate on developing advanced analysis algorithms, machine learning approaches, and statistical modeling methods to extract comprehensive insights from acquired data. Integrating single-molecule imaging data with other techniques such as genomics, proteomics, or structural biology will provide a holistic understanding of biological processes, facilitating the correlation of molecular behavior with higher order cellular functions.

Expanding fluorescence-based single-molecule imaging to in vivo settings and dynamic cellular environments poses significant challenges. Factors like autofluorescence, scattering, motion artifacts, and physiological conditions present formidable hurdles. Future directions should explore strategies to address these obstacles, including the design of biocompatible fluorophores, advanced imaging approaches to mitigate tissue scattering, and imaging techniques capable of capturing real-time dynamics in living systems.

One of the first biological applications of single-molecule TIRF was published in 1995 [112]. The fluorophores (Cy3 and Cy5), the labeling strategy (cysteine–maleimide), and the type of camera (CCD) used in the first application are still often used today [112]. However, these fluorophores and labeling strategies do not work for every biological system. Now, researchers have more choices (i.e., passivation, conjugation, immobilization, and analysis strategies) to apply single-molecule TIRF to almost any biological system. Certain steps still pose challenges, notably the site-specific labeling of large proteins and protein complexes. Nevertheless, the expanded options, at the very least, provide ways to address these complexities. For example, the unnatural amino acid conjugation strategy enables the application of single-molecule TIRF to large biomolecules such as the 2.5 MDa 26S proteasome [54]. Additionally, the liposome immobilization strategy indirectly constrained the location of the protein to improve the folding/unfolding measurements of proteins [46]. From a survey of the recent literature on biological applications, one of the last remaining areas to improve is the ability to visualize diverse protein post-translational modifications. Future studies should aim to develop fluorescent reporters for glycosylation, phosphorylation, and methylation.

Lab-on-a-chip devices offer researchers a powerful tool to scale up the application of single-molecule fluorescence imaging. These microfluidic platforms provide unprecedented capabilities, enabling precise fluid flow and microenvironmental control, leading to enhanced signal-to-noise ratios and improved data quality. Moreover, the automation and design of lab-on-a-chip devices have the potential to substantially enhance data collection throughput, potentially accommodating hundreds of conditions and samples within one device. To enable these single-molecule devices, researchers should simplify the pipeline of in vitro single-molecule fluorescence imaging and tailor the design of the devices.

## Figures and Tables

**Table 1:** Overview of biological applications.

Target Biomolecule(s)	Fluorophore Labeling	Fluorophores	Biotin Conjugation	Surface	Analysis Software	Results	Camera	Ref
*Conformational Dynamics*								
*Streptococcus pyogenes* CRISPR Cas9	Cysteine–maleimide	Cy3 LD750	Biotinylated DNA	PEG	Custom	Cas9′s HNH domain exhibits dynamics coupled with non-target strand cleavage	EMCCD	Wang [51]
*Saccharomyces cerevisiae* 26S Proteasome	Cysteine–maleimide Unnatural amino acid “click” chemistry	Cy3 LD555 LD655	AviTag fusion reacted with BirA in vitro	PEG	SPARTAN	Ubiquitin chain binding to the 26S proteasome reduces the rate of conformational transitions	EMCCD	Jonsson [54]
*Mus musculus* metabotropic glutamate receptor 2 and 3	Unnatural amino acid “click” chemistry	Cy3 Cy5	Commercial biotinylated antiFLAG antibody	PEG	smCamera software	Metabotropic glutamate receptor 2 displays four sequential conformational states	EMCCD	Liauw [63]
*Protein Folding*								
*Escherichia coli* adenylate kinase	Cysteine–maleimide	Alexa 488 Atto 590	Biotinylated phosphoethanolamine	Lipids	Custom MATLAB	folding of adenylate kinase involves at least 6 states with sequential and non-sequential transitions	SPAD	Pirchi [46]
*Protein Interactions*								
*Saccharomyces cerevisiae* cofilin on actin	Cysteine–maleimide	Alexa 488 Alexa 647	Biotinylated actin	Tween 20	TrackMate MATLAB ImageJ	Cofilin clusters grow 2 times faster towards actin’s pointed end versus barbed end	EMCCD	Bibeau [87]
Bovine β-arrestin1	Cysteine–maleimide	LD555p LD655	Strep-tag fusion	PEG	SPARTAN	β-arrestin1 tail displacement by phosphorylated C-terminal receptor requires GPCR agonist	sCMOS	Asher [58]
*S. cerevisiae* Histone and SWR1	Cysteine–maleimide	Cy3 Cy5 Cy7	Biotinylated DNA	PEG	Custom MATLAB	H2A remains in complex with SWR1–nucleosome complex for tens of seconds after H2A.Z displacement	EMCCD	Poyton [60]
Release factor complex (RFC)	Lysine hydroxysuccinimide (NHS) ester	Cy3 Atto 647	mRNA biotinylated at 3′ end	PEG	ImageJ Python	Ataluren, a translation readthrough-inducing drug, acts as a competitive inhibitor	EMCCD	Huang [88]
sRNA ChiX sRNA DsrA *Escherichia coli* Hfq sRNA chaperone	5′ sRNA-free primary amine-NHS ester	Cy3 Cy5	AviTag fusion biotinylated by endogenous BirA	Tween 20	Imscroll in MATLAB	Sometimes two sRNAs can stably bind to Hfq. Most replacement occurs when a strongly competitive sRNA, ChiX, replaces a moderately competitive sRNA, DsrA.	EMCCD	Roca [49]
*Protein post-translational*								
Human Ubc13 E2 ubiquitin ligase	Cysteine–maleimide	Cy3B Alexa 647	AviTag fusion reacted with BirA in vitro	PEG	Interactive data language (IDL)	Ubiquitin transfer proceeds from high FRET signal corresponding to the closed conformation of ubc13	EMCCD	Branigan [50]
Human anaphase-promoting complex E3 ubiquitin ligase	Cysteine–maleimide	Alexa 488 DyLight 550 Alexa 647	Intein-mediated protein ligation (IPL) of biotin-containing peptide to the C terminus	PEG	Custom MATLAB	Anaphase-promoting complex displays biphasic activity	EMCCD	Lu [56]

## Data Availability

No new data were created in this work.
